# Editorial: Artificial intelligence in neurosurgical practices: current trends and future opportunities

**DOI:** 10.3389/fneur.2026.1925792

**Published:** 2026-07-31

**Authors:** Andrea Bianconi, Marc Hohenhaus, Fabio Cofano, Mario Giordano, Santiago Cepeda, Diego Garbossa

**Affiliations:** 1 Department of Neuroscience, Rehabilitation, Ophthalmology, Genetics, Maternal and Child Health (DINOGMI), University of Genova, Genova, Italy; 2 Neurosurgery Unit, IRCCS Azienda Ospedaliera Metropolitana (AOM) Liguria, Genova, Italy; 3 Department of Neurosurgery, Medical Center University of Freiburg, Freiburg im Breisgau, Germany; 4 Neurosurgery Unit, Department of Neuroscience, University of Turin, Turin, Italy; 5 Department of Neurosurgery, International Neuroscience Institute, Hannover, Germany; 6 Department of Neurosurgery, Río Hortega University Hospital, Valladolid, Spain

**Keywords:** artificial intelligence, clinical translation, multimodal data integration, predictive analytics, risk stratification

## Introduction

Artificial intelligence (AI) has moved from a topic of academic curiosity to a structural component of neurosurgical research and, increasingly, of clinical workflows. Less than a decade after the first systematic reviews compared machine learning models against the judgement of expert neurosurgeons (1), the scope of inquiry has broadened from isolated proof-of-concept algorithms to multimodal, patient-facing systems spanning the entire surgical journey. The 12 contributions gathered in this Research Topic reflect that broadening: they originate from centers across Asia, Europe, Africa, and North America, draw on data ranging from national administrative registries to intraoperative flow cytometry and smartphone sensors, and together sketch a field that is maturing methodologically while still confronting the translational hurdles that separate a validated model from a trusted clinical tool.

A first cluster of contributions concerns image-based diagnosis and lesion characterization, an area benchmarked against multi-institutional efforts like the Brain Tumor Segmentation (BraTS) challenges and spanning preoperative segmentation, postoperative tumor-burden quantification, PET analysis, and synthetic image generation. Deep-learning pipelines have been validated for pre and postoperative brain tumor segmentation (2, 3), extended to meningioma and metastasis via meta-transfer learning (4), applied to PET delineation (5), and used to synthesize PET images from MRI (6). Against this backdrop, backbones (VGG-19) or lightweight detectors (YOLOv10n) improves both the accuracy and the speed of brain tumor segmentation on MRI. Netshamutshedzi et al. backbones (VGG-19) or lightweight detectors (YOLOv10n) improves both the accuracy and the speed of brain tumor segmentation on MRI. systematically reviewed hybrid machine learning architectures and found that combining classical classifiers such as support vector machines with convolutional backbones (VGG-19) or lightweight detectors (YOLOv10n) improves both the accuracy and the speed of brain tumor segmentation on MRI. Sanker et al. extended this picture to low-grade gliomas specifically, mapping how AI now supports tumor segmentation, hyperspectral-imaging-based tissue discrimination, and intraoperative neuronavigation, while cautioning that the evidence base, drawn from only eight eligible studies, remains preliminary. Koriyama et al. pushed the diagnostic question into the operating room, integrating preoperative imaging with intraoperative flow cytometry to predict the molecular subtype of gliomas in real time, reporting 76% accuracy and an area under the curve approaching 0.90, and prototyping a decision-support application for the surgical team.

A second, related group of studies turns from characterizing the lesion to forecasting what will happen to the patient, as cataloged by broad reviews of machine-learning outcome-prediction and preoperative-planning models (7, 8), an ambition pursued across vascular, oncological, and traumatic neurosurgery alike (9, 10). Within this Research Topic, using a national registry of more than 25,000 admissions, Wu et al. trained 12 machine learning models, the best being CatBoost (area under the curve above 0.90 on both internal and hold-out validation), to anticipate prolonged hospitalization and costs after subarachnoid hemorrhage, identifying hydrocephalus, vasospasm, and mechanical ventilation as key drivers. Hong et al. fused clinical variables with radiomics and deep-learning features to predict malignant cerebral edema after thrombectomy, reaching an area under the curve above 0.92 and outperforming the ACORNS scale, while He et al. applied SHapley Additive exPlanations (SHAP)-interpreted XGBoost models, with areas under the curve of 0.92 in training and 0.81 in external validation, to 6-month recovery after hypertensive hemorrhage, again converging on hematoma volume and physiological severity as the dominant predictors. Lehmann et al. add a different register to this prognostic effort, showing that large language models such as GPT-4 can estimate short-term survival after decompressive hemicraniectomy with modest but statistically meaningful accuracy, especially once prompts are enriched with current guideline content, although functional outcome proved harder to predict. Read together, these four studies trace a continuum from interpretable tabular models to multimodal fusion to generative AI, converging on risk stratification clinicians can interrogate.

Functional neurosurgery is represented by Bangash et al., whose systematic review of 15 studies and 352 patients on machine learning for seizure-onset-zone localization in drug-resistant epilepsy found that personalized models consistently outperformed generic ones, with the strongest approaches exceeding 90% sensitivity and specificity, while also identifying heterogeneous acquisition protocols as the field's main obstacle to cross-study comparison.

A further pair of contributions extends AI-enabled tools beyond image analysis and outcome modeling into surgical guidance and recovery monitoring, often by repurposing devices patients already own. Ye et al. showed that a smartphone-based augmented-reality application, paired with refined reference markers, can localize small intracranial lesions nearly as accurately as conventional neuronavigation, in roughly half the time and at a fraction of the cost, a proposition particularly relevant to resource-limited settings. Feurer et al. moved the smartphone from the operating theater to the patient's pocket after discharge, using passively collected GPS and accelerometer data to characterize recovery trajectories after cervical myelopathy surgery and finding meaningful correlations with established patient-reported outcome measures. Both studies suggest that some of the most accessible opportunities for AI in neurosurgery may not require specialized hardware.

Two broader contributions situate these studies within the field's trajectory. Yangi et al. systematically reviewed 181 studies across six neurosurgical subspecialties and documented how deep learning applications have grown markedly since 2019, concentrating on functional neurosurgery (notably deep-brain-stimulation and nuclei targeting), neuro-oncology (chiefly tumor segmentation and classification), and image-guided navigation within general neurosurgery. Huang complements this with a forward-looking narrative synthesis, framing the field's trajectory as a progression from simple severity scores toward predictive analytics, reporting Dice scores around 0.82–0.84 for tumor segmentation and areas under the curve of 0.80–0.90 for outcome forecasting across the studies it surveys, and looking ahead toward federated learning, generative AI, and continuous-learning ecosystems, while insisting throughout that AI is best understood as a cognitive collaborator rather than a replacement for the neurosurgeon.

That framing also captures the challenges running through nearly every contribution to this Research Topic. Interpretability is a recurring concern, addressed through SHAP-based explanations in several studies and explicit calls for explainable AI in others; external, multicentric validation remains the exception, with most models still trained and tested within single institutions; and the regulatory and medico-legal landscape is evolving more slowly than the technology itself. As Patel et al. (11) note for United States practice, AI-enabled tools for segmentation, aneurysm detection, and spinal navigation are increasingly reaching regulatory clearance, yet persistent black-box behavior and unresolved liability questions mean approval alone does not guarantee clinical trust.

Read together, these contributions suggest that the next phase of AI in neurosurgery will be defined less by isolated algorithmic breakthroughs than by the field's ability to validate models across institutions and populations, make their reasoning legible to clinicians and regulators, and favor low-cost, widely accessible solutions that integrate into everyday practice rather than specialized research centers alone. Prospective, multicenter registries; federated learning frameworks that let models learn across hospitals without centralizing sensitive data; and closer collaboration between neurosurgeons, data scientists, and regulators stand out as concrete opportunities ahead. We hope this Research Topic offers both a snapshot of where the field stands today and a useful map for the work that lies ahead, and we thank the authors and reviewers whose contributions made it possible.
